# The impact of organizational commitment on job performance in primary healthcare: a motivation internalization perspective

**DOI:** 10.3389/fpubh.2025.1685420

**Published:** 2025-10-01

**Authors:** Shichao Zhao, Tao Wang, Shanshan Luo, Yuequn Mi, Ying Wang, Huifen Ma, Xiaolin Wei

**Affiliations:** ^1^School of Public Administration, Shandong Normal University, Jinan, China; ^2^Department of Human Resources, Jinan Central Hospital, Jinan, China; ^3^School of Political Science and Public Administration, Shandong University, Qingdao, China; ^4^School of Management, Shandong University of Traditional Chinese Medicine, Jinan, China; ^5^School of Medical Management, Shandong First Medical University, Taian, China; ^6^Dalla Lana School of Public Health, University of Toronto, Toronto, ON, Canada

**Keywords:** organizational commitment, job performance, motivation internalization, primary healthcare workers, self-determination theory

## Abstract

**Introduction:**

Primary healthcare workers (PHCWs) are crucial to the healthcare system, as they directly impact the delivery of essential health services. Their job performance is influenced by various types of organizational commitment, but the effects of these commitments are not fully understood. This study aims to explore how four types of organizational commitment (affective, normative, economic, and opportunity) affect job performance among PHCWs, using Self-Determination Theory to examine motivation internalization as a mediating factor.

**Methods:**

A cross-sectional survey of 870 PHCWs from 38 primary healthcare institutions was conducted. Hierarchical regression analysis was used to explore the relationships between commitment types, motivation internalization, and job performance.

**Results:**

Affective and normative commitments positively predicted job performance, with motivation internalization partially mediating this relationship. Opportunity commitment negatively predicted job performance, mediated by reduced motivation internalization. Economic commitment showed no significant effect on either motivation internalization or job performance.

**Discussion:**

The impact of organizational commitment on job performance is shaped by its motivational quality. Strengthening affective and normative commitments through supportive incentive strategies can enhance PHCWs’ performance in primary healthcare settings.

## 1 Introduction

Ensuring a stable and motivated primary healthcare workforce remains a persistent challenge in many low- and middle-income countries. In China, this issue is particularly severe: high turnover among primary health care workers (PHCWs) continues to undermine the accessibility and quality of community-based services (1–4). Despite ongoing policy efforts, retention strategies have primarily focused on whether PHCWs remain, rather than why they remain (5). Yet remaining in one’s role does not necessarily imply strong work motivation, as some PHCWs may stay primarily due to external constraints rather than volition, potentially undermining both job performance and long-term workforce stability (6). In this context, understanding not just how many PHCWs remain, but what drives their decision to remain is increasingly important for cultivating an engaged and sustainable primary healthcare workforce.

Organizational commitment offers a valuable perspective on the motivational bases of long-term attachment, as it reflects a sustained psychological bond between individuals and their organization and helps explain why they choose to remain (7). According to Meyer and Allen’s framework, organizational commitment consists of three dimensions (8). Affective commitment involves an employee’s emotional attachment to and identification with the organization. Normative commitment reflects a sense of moral obligation to remain with the organization. Continuance commitment refers to the perceived costs associated with leaving. Subsequent research has further refined continuance commitment into two subcomponents: economic commitment, which emphasizes financial dependence as the primary reason for remaining, and opportunity commitment, which stems from a perceived lack of better job alternatives (9, 10). This refinement has led to a four-dimensional conceptualization of organizational commitment, which has been applied in subsequent studies to examine the distinct effects of economic and opportunity commitment on work outcomes (11) and to analyze their underlying motivational bases and consequences (9). Together, these dimensions reflect different motivational bases and shape PHCWs’ work experiences in different ways. Recognizing these variations helps explain why some PHCWs remain actively engaged in their roles, while others, though equally retained, contribute only minimally.

While a substantial literature has investigated the relationship between organizational commitment and job performance, findings have varied considerably depending on the type of commitment (11–13). Affective commitment is consistently associated with higher performance. Normative commitment is generally positively related to performance, though its effects are often modest (14). By contrast, continuance commitment (including economic and continuous dimensions) frequently correlates weakly or even negatively with performance (15, 16). Despite the accumulated evidence linking organizational commitment to job performance, few studies have explored how different commitment types exert their effects (17–19). To address this gap, this study introduces Self-Determination Theory (SDT) as a theoretical framework to examine the motivational mechanisms through which different types of commitment influence job performance (20, 21). SDT conceptualizes motivation as a continuum ranging from controlled form to full autonomy, with more autonomous forms consistently linked to better performance, persistence, and well-being (22–24). The shift from controlled to autonomous motivation is known as motivation internalization—a core process whereby external regulations are gradually integrated into one’s sense of self (21). According to SDT, motivation internalization is facilitated when individuals experience support for three basic psychological needs: autonomy, competence, and relatedness (22).

**Figure 1:**
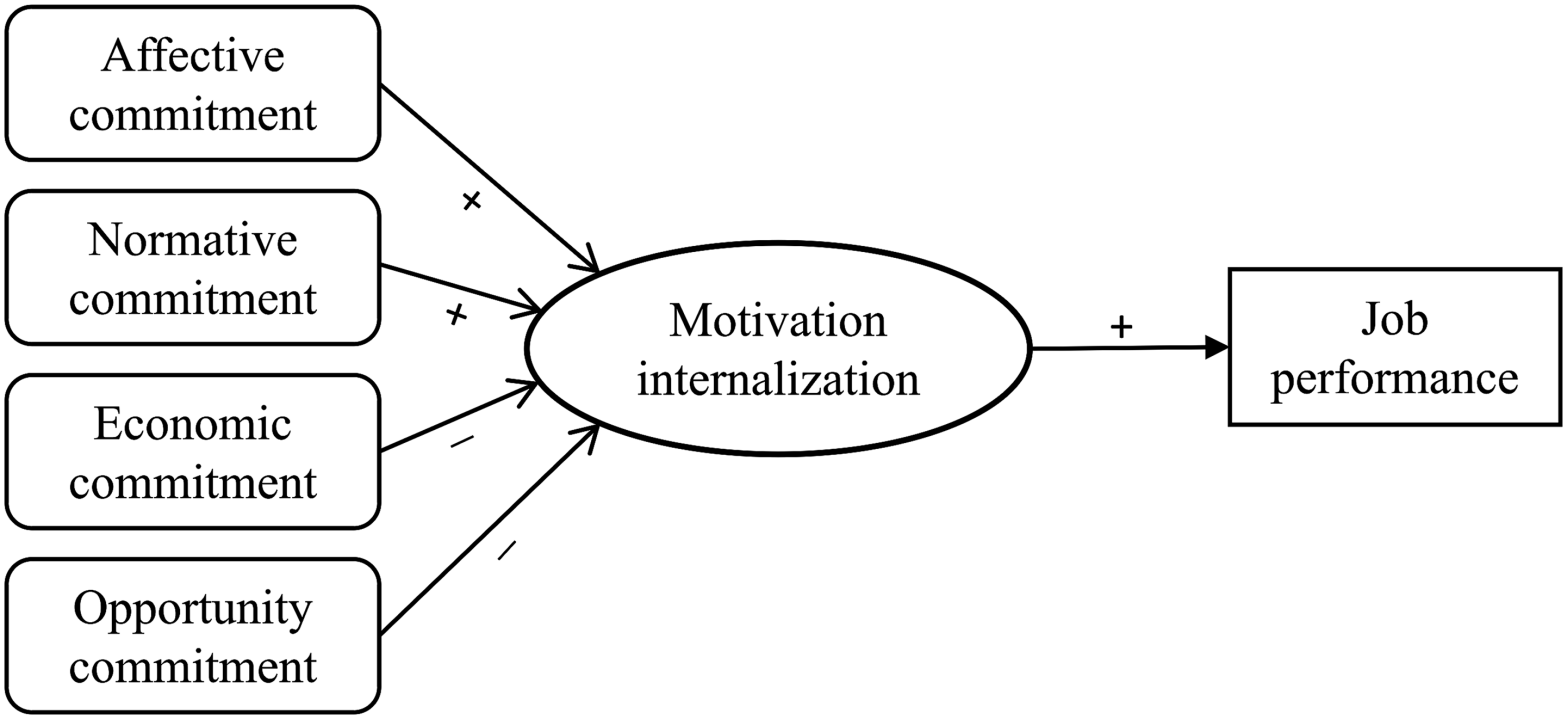
Conceptual model illustrating hypothesized relationships between commitment types, motivation internalization, and job performance.

Drawing on SDT, we posit that each commitment type influences motivation internalization by supporting or frustrating the satisfaction of basic psychological needs. Affective commitment, which reflects emotional attachment to the organization, plays a key role in satisfying the need for relatedness—by fostering a sense of belonging, trust, and identification with colleagues and patients. This relational embeddedness can promote motivation internalization (8). Normative commitment, although obligation-based, may also support internalization if the perceived obligations are self-endorsed and aligned with personal values, thereby satisfying the need for autonomy (25). In contrast, opportunity and economic commitment are typically rooted in external constraints—such as limited alternatives or financial dependence—which may frustrate the need for autonomy and, in some cases, competence. As a result, these forms of commitment are likely to impede the internalization process (26). Moreover, prior research grounded in SDT has consistently shown that more autonomous forms of motivation are linked to higher levels of job performance (23). Since motivation internalization reflects the degree to which autonomous motivation dominates work behavior (27), we expect it to positively predict job performance. Based on the above reasoning, we propose a conceptual model (Figure 1) in which the four commitment dimensions influence job performance through their different effects on motivation internalization.

This study empirically tests the proposed model based on survey data from PHCWs in Shandong Province, China. It offers a theoretical explanation for why equally committed PHCWs may perform differently, by clarifying how distinct types of commitment differentially influence motivation internalization. The findings also offer practical implications for improving provider retention and enhancing service delivery in the primary healthcare context.

## 2 Methods

### 2.1 Study design and sampling

This cross-sectional study was conducted in 2021 among PHCWs in Shandong Province, China, using a structured questionnaire. A multistage cluster sampling strategy was adopted to ensure geographic and economic representativeness. Three cities (Qingdao, Dongying, and Zaozhuang) were first selected based on regional diversity, followed by the random selection of four districts or counties within each city. Subsequently, 3–4 primary health institutions were chosen per district or county, yielding a total of 38 institutions (18 community health service centers and 20 township hospitals). All PHCWs on duty during the survey period were invited to participate.

A total of 870 valid questionnaires were returned, yielding a high response rate of 92.3%. The sample comprised 193 males (22.2%) and 677 females (77.8%). Participants ranged in age from under 30 (21.4%) to 50 years and older (8.6%), with the largest group aged 40–49 (37.8%). In terms of professional roles, 40.3% were physicians, 29.5% nurses, 15.8% medical technicians, 8.3% public health staff, and 6.1% administrative or logistical personnel.

### 2.2 Measure

#### 2.2.1 Organizational commitment

Organizational commitment was measured using an adapted scale developed for PHCWs (28), encompassing four dimensions: affective, normative, economic, and opportunity commitment. Each dimension was assessed with three items (12 items in total) on a 5-point Likert scale (1 = strongly disagree, 5 = strongly agree), yielding scores from 3 to 15, with higher scores indicating stronger commitment. The Cronbach’s alpha coefficients for the four dimensions were 0.903 for affective commitment, 0.862 for normative commitment, 0.849 for economic commitment, and 0.758 for opportunity commitment, indicating acceptable internal consistency. Prior research provides structural validity based on factor analysis (15) and convergent, discriminant, and criterion validity across established antecedents and outcomes (11).

#### 2.2.2 Motivation internalization

Motivation internalization was assessed based on the structure of work motivation outlined in SDT. Work motivation was measured using an adapted version of the Chinese Work Motivation Scale for Healthcare Workers (27, 29). The scale comprises 18 items covering five dimensions: amotivation (3 items, Cronbach *α* = 0.797), external regulation (6 items, Cronbach α = 0.754), introjected regulation (3 items, Cronbach α = 0.887), integrated regulation (3 items, Cronbach α = 0.876), and intrinsic motivation (3 items, Cronbach α = 0.769). All items were rated on a 7-point Likert scale (1 = completely disagree, 7 = completely agree), with higher scores indicating greater intensity of motivation. Prior validation of the MWMS has demonstrated robust structural validity and cross-cultural measurement invariance across languages and countries, supporting its applicability in diverse contexts (27). In addition, a recent study among Chinese primary healthcare workers employed the adapted Chinese version, further supporting its cross-cultural applicability (29).

The degree of motivation internalization was assessed using the Self-Determination Index (SDI), which aggregates the five motivation subscales by assigning differential weights that reflect their relative position on the autonomy continuum, ranging from amotivation (the least autonomous) to intrinsic motivation (the most autonomous) (30, 31). The formula is as follows:


$$\begin{array}{c} \mathrm{SDI}=3\cdot \text{Intrinsic Motivation}+1.5\cdot \text{Integrated Regulation Motivation}\\ -1\cdot \text{Introjected Motivation}-2\cdot \text{External Motivation}\\ -3\cdot \text{Amotivation} \end{array}$$

#### 2.2.3 Job performance

Job performance was measured using a 10-item scale developed by Zhao et al. specifically for PHCWs in China. The same study reported a multidimensional structure with acceptable global fit indices, indicating structural validity (32). The scale assesses three dimensions of job performance: task performance, contextual performance, and learning performance. Each item was rated on a 7-point Likert scale (1 = completely disagree, 7 = completely agree), and the total score, which ranged from 10 to 70, was used as the outcome variable in this study, with higher scores reflecting better performance. The scale demonstrated excellent internal consistency, with Cronbach’s alpha coefficient of 0.909.

#### 2.2.4 Data analysis

Descriptive statistics and Pearson correlation analyses were conducted to examine basic patterns and associations among organizational commitment, motivation internalization, and job performance. To test the mediating role of motivation internalization, we followed Baron and Kenny’s three-step approach (33). All analyses were performed using SPSS version 22.0, with statistical significance set at *p* < 0.05.

## 3 Results

### 3.1 Descriptive statistics

Descriptive statistics showed notable variation across the four dimensions of organizational commitment among PHCWs. On average, affective commitment received the highest score (M = 3.78, SD = 0.86), followed by normative commitment (M = 3.55, SD = 0.89). In contrast, economic commitment (M = 2.93, SD = 1.09) and opportunity commitment (M = 2.89, SD = 1.04) were relatively lower. Additionally, the average scores for motivation internalization and job performance were 5.40 (SD = 1.02) and 5.02 (SD = 0.99), respectively.

### 3.2 Correlation analysis

Significant correlations were observed among organizational commitment, motivation internalization, and job performance based on Pearson analysis (Table 1). Among the four commitment dimensions, affective commitment (*r* = 0.488, *p* < 0.01) and normative commitment (*r* = 0.387, *p* < 0.01) demonstrated moderate positive correlations with motivation internalization. Economic commitment was weak but significantly associated (*r* = 0.086, *p* < 0.05), whereas opportunity commitment showed a small negative correlation (*r* = −0.078, *p* < 0.05). As anticipated, motivation internalization was strongly associated with job performance (*r* = 0.499, *p* < 0.01). Affective (*r* = 0.460, *p* < 0.01) and normative commitment (*r* = 0.391, *p* < 0.01) were also positively associated with job performance. While economic commitment showed a weak positive correlation (*r* = 0.094, *p* < 0.01), opportunity commitment was not significantly associated with job performance (*r* = −0.062, *p* > 0.05).

**Table 1:** Correlations among organizational commitment, motivation internalization, and job performance.

Variables	*Mean ± SD*	Affective commitment	Normative commitment	Economic commitment	Opportunity commitment	Motivation internalization	Job performance
Affective Commitment	3.78 ± 0.86	1					
Normative Commitment	3.55 ± 0.89	0.696^**^	1				
Economic Commitment	2.93 ± 1.09	0.165^**^	0.218^**^	1			
Opportunity Commitment	2.89 ± 1.04	0.003	0.155^**^	0.551^**^	1		
Motivation Internalization	5.40 ± 1.02	0.488^**^	0.387^**^	0.086^*^	−0.078^*^	1	
Job Performance	5.02 ± 0.99	0.460^**^	0.391^**^	0.094^**^	−0.062	0.499^**^	1

Significant differences are indicated by *p < 0.05 and **p < 0.01.

### 3.3 Regression-based mediation analysis

Hierarchical regression was conducted to test the hypothesized mediation model (Table 2). In Model 1, affective commitment (*β* = 0.32, *p* < 0.001), normative commitment (*β* = 0.16, *p* < 0.01), and opportunity commitment (*β* = −0.12, *p* < 0.01) significantly predicted job performance, while economic commitment showed no significant association (*β* = 0.04, *p* > 0.05).

**Table 2:** Regression analysis of organizational commitment, motivation internalization, and job performance.

Variable	Model 1 (Y = Job performance, *β*)	Model 2 (Y = Motivation internalization, *β*)	Model 3 (Y = Job performance, *β*)
Affective Commitment	0.32^**^	0.37^**^	0.19^**^
Normative Commitment	0.16^**^	0.12^**^	0.12^**^
Economic Commitment	0.04	0.07	0.02
Opportunity Commitment	−0.12^**^	−0.14^**^	−0.07^*^
Motivation Internalization			0.34^**^
Gender (ref: Female)			
Male	0.04	−0.03	0.05
Age (ref: <30)			
30 ~ 39	0.05	0.00	0.05
40 ~ 49	0.04	−0.00	0.04
≥50	0.02	0.05	0.01
Profession (ref: Administrative)			
Physician	0.05	0.06	0.03
Nurse	0.02	−0.00	0.02
Public health	0.04	−0.01	0.05
Medical technical	0.01	0.02	0.01
Education (ref: ≤ Secondary School)			
Junior College	−0.09	−0.04	−0.08
≥ Bachelor’s Degree	0.05	−0.02	0.06
Professional Title (ref: ≤ None)			
Junior	0.05	0.03	0.04
Intermediate	0.06	0.02	0.05
Senior	0.01	0.02	0.01
Institution Type (ref: Community)			
Township	0.05	0.08^*^	0.02
F	17.31^***^	17.32^***^	24.13^***^
*R*^2^	0.268	0.268	0.350

Standardized regression coefficients are reported. Control variables include gender, age, profession, education, professional title, and institution type. *p < 0.05, **p < 0.01, ***p < 0.001.

In Model 2, motivation internalization was a significant predictor of job performance (*β* = 0.34, *p* < 0.001), supporting its potential role as a mediating variable.

After adding motivation internalization in Model 3, the effects of affective, normative and opportunity commitment were attenuated to *β* = 0.19 (from 0.32), *β* = 0.12 (from 0.16), and *β* = −0.07 (from −0.12), respectively, although all three remained significant (*p* < 0.01). This pattern suggests partial mediation, whereby these types of commitment influence performance both directly and indirectly through motivation internalization. In contrast, economic commitment remained nonsignificant across all models. Specifically, the indirect effect via motivation internalization was 0.125 for affective commitment, accounting for 39.12% of its total effect; 0.042 for normative commitment (26.59%); and −0.047 for opportunity commitment (40.94%).

Bootstrapped mediation analysis (5,000 resamples) further supported the mediating effects, with all indirect effects reaching statistical significance. The bias-corrected 95% confidence intervals excluded zero for each pathway: [0.083, 0.172] for affective commitment, [0.010, 0.076] for normative commitment, and [−0.081, −0.019] for opportunity commitment, indicating robust and consistent mediation.

## 4 Discussion

This study examined how distinct dimensions of organizational commitment influence job performance among PHCWs in China. Extending prior study on the commitment–performance relationship, it found that motivation internalization acts as a critical psychological mechanism through which certain types of commitment exert their effects (34, 35).

PHCWs reported the highest scores for affective and normative commitment, suggesting that many remain due to emotional attachment and perceived obligation. The prominence of affective commitment may reflect the relational continuity and community embeddedness characteristic of primary healthcare. PHCWs often serve the same patients over long periods, which fosters interpersonal trust, professional identification, and a sense of belonging (36). As these relationships are developed and maintained through their organizational roles, the emotional bonds with the community can extend to the organization. Meanwhile, the relatively high level of normative commitment may reflect PHCWs’ recognition of their essential role as gatekeepers of community health. This recognition can reinforce a strong sense of moral duty—not only to the communities they serve, but also to the organizations that enable their work (11). In addition, given that 77.8 percent of participants were women, the relatively higher levels of affective and normative commitment observed in this sample may partly reflect gender composition. Social role accounts suggest that, on average, women endorse more communal and relationship-oriented values, which can align with stronger emotional attachment and a greater sense of obligation to the organization (36). Consistent with this possibility, meta-analytic evidence suggests that women may exhibit higher affective commitment compared to men (37), and some studies also report slightly higher normative commitment among women (38, 39).

In contrast, economic and opportunity commitment were notably lower, suggesting that most PHCWs did not feel strongly constrained by financial dependency or limited alternatives. The relatively low level of economic commitment observed in this study may be partly attributable to a broader pattern identified in previous research, which shows that PHCWs often attach greater importance to non-financial incentives—such as stability, meaning, and manageable workloads—than to monetary compensation (40). Similarly, the lower level of opportunity commitment may reflect PHCWs’ limited concern with external job mobility, possibly due to job satisfaction, perceived job security, or habituated career paths that diminish the salience of alternative options.

The findings support the hypothesized pathway in which both affective and normative organizational commitment enhance job performance by promoting motivation internalization. Notably, affective commitment exerted a stronger effect on both motivation internalization and job performance compared to normative commitment. Affective commitment, rooted in emotional attachment and value identification, aligns more closely with autonomous motivation and thus showed a stronger effect. Normative commitment, driven by obligation and social expectation, may also support internalization but tend to involve more controlled regulation (41). In the primary healthcare context, where providers maintain long-term relationships with patients and are deeply rooted in local communities, emotional bonds and moral responsibility often coexist and reinforce one another (11), as evidenced by the strong positive correlation between affective and normative commitment in our findings. Under such conditions, PHCWs with high affective and normative commitment are more likely to view organizational goals as personally meaningful rather than imposed. This shift from compliance to identification fosters sustained, self-congruent motivation, which in turn supports sustained job performance.

Opportunity commitment was found to negatively affect both motivation internalization and job performance. Some PHCWs may remain in their position not by choice, but due to institutional constraints (42). This form of “passive retention” may undermine perceived autonomy and create a psychologically restrictive state. As suggested by SDT, such perceived external control reduces the likelihood of internalizing organizational goals, leading to more controlled forms of motivation and, ultimately, diminished job performance (21). Economic commitment was originally hypothesized to suppress performance by reducing the degree of motivation internalization, but the results showed no significant effect. This suggests that while financial dependence may explain continued employment, it neither energizes nor impairs motivation in this context. One possible reason is that stable income and job security are perceived as baseline conditions rather than active drivers of effort. This interpretation is consistent with Herzberg’s two-factor theory, which categorizes such factors as necessary to avoid dissatisfaction but insufficient to promote high motivation (43). In this sense, economic commitment may represent a more neutral form of attachment in terms of motivational quality: it does not facilitate internalization, but it may also avoid triggering controlled regulation.

This study advances understanding of organizational commitment by empirically comparing four distinct dimensions: affective, normative, opportunity, and economic, and uncovering the motivational mechanism through which they influence job performance. Drawing on SDT, the study confirms that different types of commitment influence motivation internalization to varying degrees. This finding underscores that the motivational quality of commitment plays a critical role in shaping behavioral effectiveness. Moreover, the study lends empirical support to a four-dimensional conceptualization of organizational commitment by distinguishing between economic and opportunity commitment. Though commonly conceptualized as a single dimension known as continuance commitment (8), these two dimensions exhibited distinct effects on motivation and performance, underscoring the theoretical and practical value of treating constraint-based commitment as a multidimensional construct.

These findings have some implications for human resource incentive policies in primary healthcare. A primary focus is to strengthen affective and normative commitment, as they support motivation internalization and improved job performance. To this end, institutions should foster psychologically supportive work environments by aligning performance management systems with autonomy and recognition, while also reinforcing daily managerial practices such as team communication, peer mentoring, and participatory decision-making. Furthermore, the negative effects associated with opportunity commitment underscore the risks of constraint-based retention, often stemming from institutional limitations such as restricted mobility or narrow promotion pathways. To address this, primary healthcare institutions should consider expanding horizontal development pathways, such as offering cross-institution rotations or diversified professional tracks. In addition, strengthening collaboration and resource sharing with higher-level hospitals can provide PHCWs with expanded professional development opportunities, without requiring formal job transfer.

Several limitations should be acknowledged. First, although the model is conceptually grounded in SDT and supported by empirical associations, the cross-sectional design limits causal interpretations of the observed relationships. In particular, self-reported measures may be subject to certain biases, such as social desirability. To strengthen causal inference and reduce potential bias, future studies should adopt longitudinal or experimental designs and incorporate more objective indicators of job performance, such as institutional or administrative records. Second, although Shandong Province shares many institutional features with other regions in China, the findings may not generalize to countries with primary healthcare systems and sociocultural environments that differ structurally from China’s. Differences in institutional arrangements, workforce policies, and cultural values may shape how organizational commitment and motivation internalization influence job performance. Future research should therefore include cross-cultural or multinational samples to enhance the external validity of findings. Third, this study primarily focuses on individual-level psychological mechanisms, without sufficiently considering institutional or organizational-level factors that may also shape job performance. Elements such as promotion systems, compensation structures, leadership styles, and governance arrangements could play a significant role in shaping how organizational commitment affects motivation internalization and job performance. Future research should therefore integrate organizational and policy-level variables, and ideally adopt multilevel research designs, to provide a more comprehensive and context-sensitive understanding of commitment dynamics in primary healthcare.

## 5 Conclusion

This study examined how different types of organizational commitment influence job performance among PHCWs in China, focusing on the mediating role of motivation internalization. The findings show that affective and normative commitment enhance both motivation internalization and job performance, while opportunity commitment has a negative influence and economic commitment shows no significant effect. Drawing on SDT, the study demonstrates that the impact of organizational commitment depends not just on its presence, but on the motivational quality of different commitment types—specifically, the extent to which they support internalized motivation. These insights highlight the managerial value of fostering supportive work environments that enhance affective and normative commitment and reduce opportunity commitment.

## Data Availability

The raw data supporting the conclusions of this article will be made available by the authors, without undue reservation.
